# Draft Genome Sequence of “*Candidatus* Moanabacter tarae,” Representing a Novel Marine Verrucomicrobial Lineage

**DOI:** 10.1128/MRA.00951-18

**Published:** 2018-10-18

**Authors:** Julian Vosseberg, Joran Martijn, Thijs J. G. Ettema

**Affiliations:** a Department of Cell and Molecular Biology, Science for Life Laboratory, Uppsala University, Uppsala, Sweden; b Theoretical Biology and Bioinformatics, Department of Biology, Utrecht University, Utrecht, The Netherlands; Georgia Institute of Technology

## Abstract

The Tara Oceans Consortium has published various metagenomes of marine environmental samples. Here, we report a contig of 2.6 Mbp from the assembly of a sample collected near the Marquesas Islands in the Pacific Ocean, covering a nearly complete novel verrucomicrobial genome. We propose the name “*Candidatus* Moanabacter tarae” for the corresponding bacterium.

## Abstract (precis)

The Tara Oceans Consortium has published various metagenomes of marine environmental samples. Here, we report a contig of 2.6 Mbp from the assembly of a sample collected near the Marquesas Islands in the Pacific Ocean, covering a nearly complete novel verrucomicrobial genome.

## ANNOUNCEMENT

*Verrucomicrobia* is a bacterial phylum within the PVC superphylum, which additionally includes, *inter alia*, *Planctomycetes* and *Chlamydiae* (1). Although the phylum comprises a relatively small fraction of the marine bacterioplankton (2), its widespread occurrence tentatively points to significant ecological roles (3). From the vast majority of prokaryotic diversity, including verrucomicrobial lineages, genomic sequences are lacking. Recent advances in cultivation-independent approaches have led to the elucidation of many novel lineages and have provided various new insights (4). A promising source for the discovery and retrieval of new genomes is the metagenomic data of ocean samples generated by the Tara Oceans Consortium, which have been made publically available (5).

The sample of interest had been collected at Tara sampling site 125 (8°54'14.35"S 142°33'58.54"W), marine epipelagic mixed layer (140 m), size fraction 0.22 to 3 μm (GenBank BioSample number ERS492926), and had subsequently been sequenced using Illumina HiSeq technology (SRA accession number ERR599156) (5). The preprocessing and assembly were performed as described previously (6). The raw sequence data were downloaded from the European Bioinformatics Institute (EMBL-EBI) (ENA accession number ERR599156). From the resulting sequencing reads, we removed adapter sequences and low-quality ends and reads using Trimmomatic v0.35 (ILLUMINACLIP, TruSeq-3-PE-2.fa:2:30:10:1:true; LEADING, 3; TRAILING, 3; MINLEN, 60; AVGQUAL, 29) (7). Assembly was performed with the meta option in SPAdes v3.7.0 (8), using *k*-mers of 21, 33, 55, and 77.

The longest contig in this assembly was 2,633,965 bp and had a GC content of 46%. Based on the presence of 131 out of 139 bacterial single-copy marker genes (9) and the genome completeness estimation method miComplete v0.2.0 (https://bitbucket.org/evolegiolab/micomplete), which takes the general distances between these marker genes into account, the contig was estimated to cover 92% of the complete genome. This genome was thereby calculated to have a length of approximately 2.85 Mbp.

**FIG 1:**
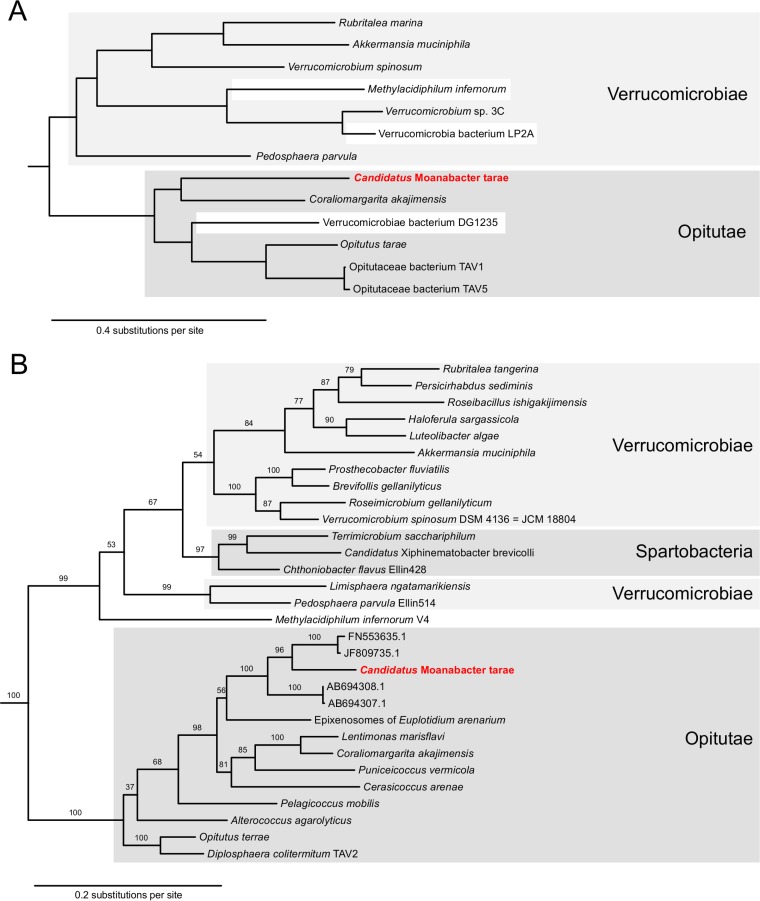
Phylogenetic position of the newly identified “*Candidatus* Moanabacter tarae” within the verrucomicrobial class *Opitutae*. (A) Placement of “*Candidatus* Moanabacter tarae” (red) within the *Verrucomicrobia* in the CheckM reference tree, which is based on 43 universal single-copy marker genes. (B) Verrucomicrobial phylogeny based on the 16S rRNA gene indicating the evolutionary relationship of “*Candidatus* Moanabacter tarae” (red) with the distinct verrucomicrobial classes. Branch labels correspond to bootstrap values. The tree was rooted with *Lentisphaerae*, *Chlamydiae*, and *Planctomycetes* (data not shown).

The contig was annotated with Prokka v1.13 (default parameters) (10). A total of 2,237 coding sequences, 48 tRNA genes, and 3 rRNA genes were predicted to be present. A BLAST search of the 16S rRNA gene recovered several uncultured marine sediment bacteria (GenBank accession numbers FN553635, JF809735, AB694308, and AB694307; 91 to 92% identity), epixenosomes of Euplotidium arenarium (87% identity), and Coraliomargarita akajimensis (86% identity) as the top hits, demonstrating the verrucomicrobial origin and novel nature of the contig. The verrucomicrobial affiliation of this new genome was confirmed by its position in a reference genome tree based on 43 marker genes (Fig. 1A), as inferred by CheckM v1.0.11 (tree command, with the predicted proteins provided as input) (11).

To more precisely infer the phylogenetic position of this novel verrucomicrobial lineage, we aligned the 16S rRNA gene with the top BLAST hits and other 16S rRNA sequences from a diverse set of *Verrucomicrobia*, *Lentisphaerae*, *Chlamydiae*, and *Planctomycetes* species, with MAFFT E-INS-i v7.050b (12) and inferred a phylogenetic tree with IQ-TREE v1.5.0a (GTR+R4 selected by the –m TESTNEW option, with 100 nonparametric bootstraps) (13). The resulting tree was in line with the BLAST and CheckM results and indicated that the organism represented by the contig was a member of a clade further consisting of marine sediment bacteria, within the verrucomicrobial class *Opitutae* (Fig. 1B).

For the newly identified marine bacterium reported here, we propose the name “*Candidatus* Moanabacter tarae,” named after the Marquesan word for ocean, “moana,” and the Tara Oceans project, with Tara aptly also being the name of a sea goddess in Polynesian mythology.

### Data availability.

The genome sequence reported here has been deposited in GenBank under the accession number CP029803.
